# Longitudinal association between maternal psychological stress during pregnancy and infant neurodevelopment: The moderating effects of responsive caregiving

**DOI:** 10.3389/fped.2022.1007507

**Published:** 2022-11-18

**Authors:** Yuyang Shi, Yudi Zhang, Qian Wei, Xuemei Ma, Yunhui Zhang, Huijing Shi

**Affiliations:** ^1^ Key Laboratory of Public Health Safety, Ministry of Education, Department of Maternal, Child and Adolescent Health, School of Public Health, Fudan University, Shanghai, China; ^2^ Key Laboratory of Public Health Safety, Ministry of Education, Department of Environmental Health, School of Public Health, Fudan University, Shanghai, China

**Keywords:** maternal psychological stress, early development, responsive caregiving, birth cohort study, *in utero* exposure

## Abstract

**Background:**

Little is known regarding the role of responsive caregiving in the association between maternal psychological stress and child neurodevelopment. We, therefore, herein investigated the relationship between maternal psychological stress during pregnancy and children's neurodevelopment with modifications in responsive caregiving.

**Methods:**

A total of 3,603 mother–child pairs were recruited from the Shanghai Maternal-Child Pairs Cohort, and we assessed maternal psychological stress using the Life Events Scale for Pregnant Women (LESPW) during early and late pregnancy. Early neurodevelopment of infants at 6 and 12 months of age was also evaluated using the Age and Stage Questionnaire, Third Edition (ASQ-3). The 2-month-old infant nursing-care questionnaire was designed based on the Five Elements of Parenting Care Framework released by the World Health Organization (WHO) and used to evaluate the levels of early responsive caregiving for infants. Multivariate logistic regression analysis was then applied to determine the association between maternal psychological stress during pregnancy and child development.

**Results:**

The suspected developmental delay rate of infants aged 6 and 12 months ranged between 13.3% and 24.5%. After adjusting for confounders, we noted that high maternal subjective events stress during early pregnancy was associated with an increased risk of suspected developmental delay in problem-solving domains at 12 months of age [adjusted OR (aOR) = 1.51; 95% confidence interval (CI), 1.09–2.20]. High general negative objective events’ stress during late pregnancy also constituted a risk factor for development in the personal–social domain at 12 months of age (aOR = 1.57; 95% CI, 1.13–2.19). Remarkably, we noted in infants with insufficient responsive caregiving that there were greater associations between the risk of general maternal negative objective events during late pregnancy and personal–social domain at 12 months of age (aOR = 2.06; 95% CI, 1.15–3.68). Similarly, there was a greater association between the risk for maternal subjective events during early pregnancy and problem-solving at 12 months of age (aOR = 1.55; 95% CI, 1.11–2.34).

**Conclusions:**

Maternal psychological stress during pregnancy was predominantly associated with suspected developmental delay in infants at 6 and 12 months of age, and these associations were modified by early responsive caregiving.

## Introduction

About 250 million children younger than 5 years of age in low-income and middle-income countries are at risk of not attaining their developmental potential (1). Although psychological stress during pregnancy has been documented to play a major role in shaping offspring socioemotional development (2–4), the findings remain equivocal. Emerging literature suggests that the impacts of maternal prenatal psychological stress on infants may depend on the degree of stress (5), type of stress exposure (6), timing of the stress experienced, and the period in which a child's neurodevelopment is measured. However, the extant literature regarding the impacts of maternal prenatal stress on an infant's early development is diffuse or entails cross-sectional studies where it is difficult to conduct comprehensive analyses of the effects of psychological stress on childhood neurodevelopment.

Responsive caregiving implies that caregivers provide appropriate responses to a child's signals (7), and it has been shown to be a potential influencing factor in child neurodevelopment. Providing sufficient responsive caregiving in early life is conducive to children's physical and mental development and the realization of early developmental potential (8, 9). Previous investigators found that sufficient responsive caregiving was conducive to the development of children's cognition, language, athletic ability, and social emotion, and it could circumvent the influence of premature birth or other adverse factors on children's physical or mental development (10). In one study, authors ascertained that insufficient responsive caregiving at 2 months of age significantly and broadly stunted infant neurodevelopmental domains, especially the personal–social domain (11). Human studies of mothers/full-term infants showed a positive association between mother–infant physical contact and multiple neurodevelopmental domains (12). Responsive caregiving has been listed as one of the five elements of the Early Childhood Development Parenting Care Framework released by the World Health Organization (WHO) at the 2016 World Health Assembly (13, 14).

Causal factors do not act uniformly under all circumstances. An effect modification (also known as interaction or heterogeneity) can be described as follows: “If the effect of an exposure (E) on an outcome (O) depends on a third variable (M), M is an effect modifier” (15). The presence of interactions is significant from a public health perspective in prevention and intervention with respect to stressors and in the identification of the most vulnerable populations (16). The presence of critical periods in early life (including prenatal and early postnatal) in child neurodevelopment (7, 17) and the potentially instrumental effects of responsive caregiving necessitate the investigation of relationships among maternal prenatal psychological stress, responsive caregiving, and child neurodevelopment in early life. However, there are relatively few extant studies on responsive caregiving for infants, and the effect modification of caregiving has not yet been studied with regard to the process of maternal psychological stress as it impacts infant neurodevelopment.

As suggested by previous studies, continuous observation based on multiple timepoints and stages is urgently needed to investigate the impacts of maternal psychological stress during different periods of pregnancy on early development of infants at different ages, as well as the role and effect of early responsive caregiving in this process. Thus, based on a prospective birth cohort, the purpose of the current study was to investigate the association between maternal psychological stress and children's neurodevelopment in infancy and to ascertain the modifying effects of responsive caregiving between maternal psychological stress and early neurodevelopment in infants.

## Methods

### Study subjects

**Figure 1:**
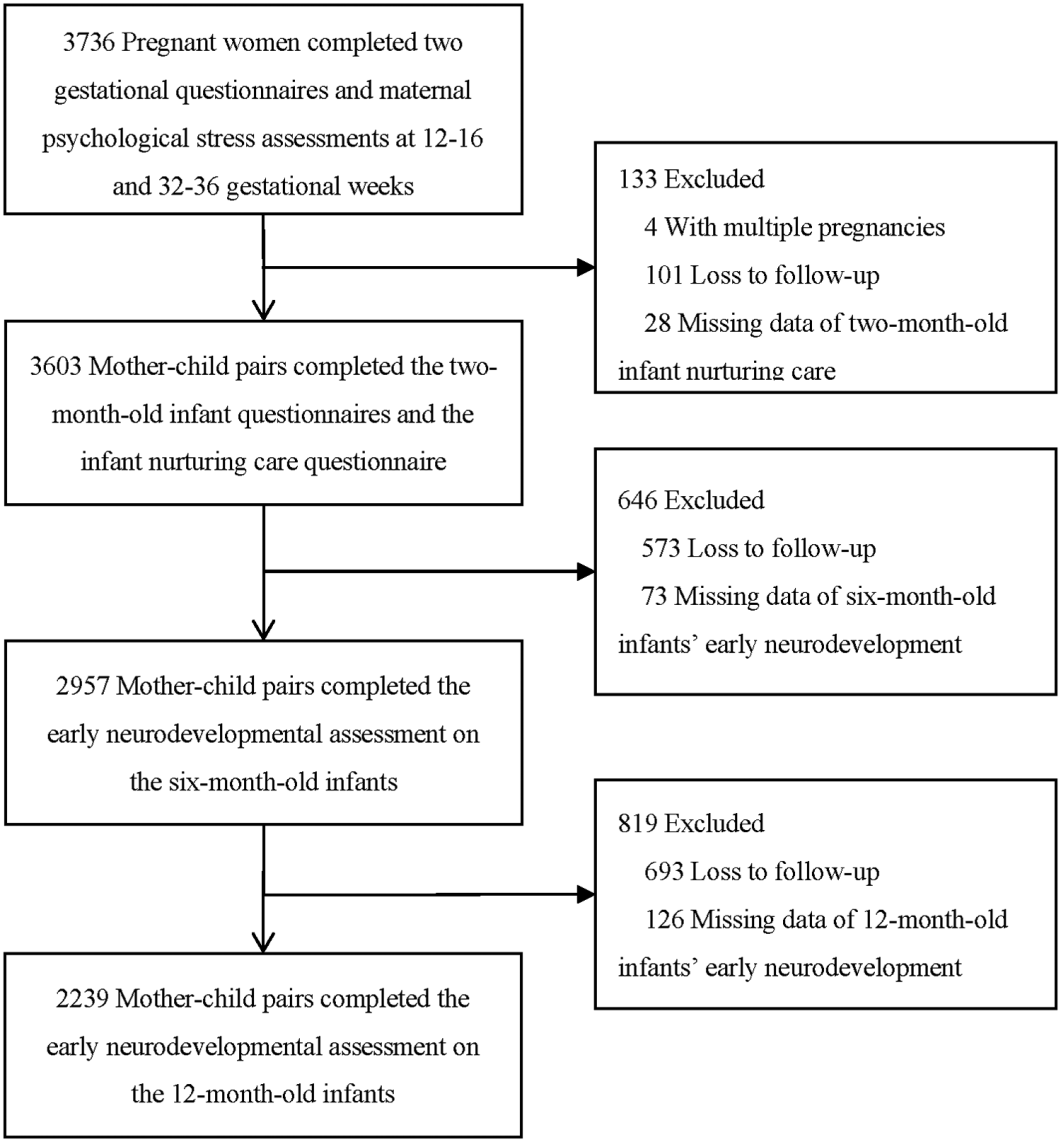
Flowchart describing the selection process for the pregnant women and the follow-up on their children.

This study was conducted as a part of the Shanghai Maternal Child Pairs Cohort (Shanghai MCPC), a large, prospective birth cohort study that began in April of 2016 (criteria and protocols for enrollment have been described previously by Ma et al.) (18). The purpose of the MCPC is to explore the influence of perinatal psychological stress, environmental pollutant exposure, and lifestyle behaviors on maternal and infant health (19, 20). We recruited eligible pregnant women from the two regional maternity hospitals of Shanghai's Pudong and Songjiang Districts at their first prenatal visit, and the samples for the present study were restricted to singleton pregnancies that resulted in a live-born infant with enrollment until June of 2019. A total of 3,736 pregnant women completed two gestational questionnaires at 12–16 and 32–36 gestational weeks. Of these cases, 3,603 mothers completed the 2-month-old infant questionnaires as well as the 2-month-old infant-nurturing care questionnaire; and we retrieved information on infant early development at the ages of 2, 6, and 12 months. Overall, the present study included 2,957 mother–child pairs who completed the early neurodevelopmental assessment on the 6-month-old infants. Among them, 2,239 mother–child pairs completed the assessment on the 12-month-old infants (a flowchart of participation is depicted in Figure 1). In order to ensure the validity of the questionnaire content adopted by the Research Institute, the questionnaire for the 2-month-old survey was completed 40–90 days after birth. This study was approved by the Ethics Committee of the School of Public Health, Fudan University (IRB number 2016-04-0587, IRB# 2016-04-0587-EX) and met the medical ethics standards of both hospitals.

### Maternal psychological stress assessments

The Life Events Scale for Pregnant Women (LESPW) was applied to assess maternal psychological stress at 12–16 weeks and 32–36 weeks during pregnancy by employing questionnaires. The LESPW is a self-rating scale used to assess perceived stress levels during pregnancy. This scale consists of 53 items on family, work, learning, and social relationship-related problems, and the events are weighted to calculate the LESPW total score. The total scores ranged from 0 to 2955, and a higher score indicated a higher level of stress during pregnancy (except for OE1, where a higher score indicated lower stress) (21). We classified maternal subjective stress (SE), positive objective stress (OE1), general negative objective stress (OE2), and severe negative objective stress (OE3) into two groups, one with high stress (equal to or above the 75th percentile) and one with low stress (below the 75th percentile) (20). The LESPW showed good reliability with a Cronbach's *α* of 0.961 for the total and 0.776, 0.880, 0.892, and 0.902 for the subscales, respectively. The correlation coefficient of criterion-related validity between the total LESPW score and the social readjustment rating scale was 0.727 (22).

### Assessment of infants’ early neurodevelopment

The Ages and Stages Questionnaire, Third Edition (ASQ-3) is a widely used, parent-reported screening measure (23, 24) that is completed when the children are 6 and 12 months (25). The ASQ-3 identifies developmental progress in five domains: communication, gross motor, fine motor, problem-solving, and personal–social skills. There are 30 items for each age group, and they are scored as yes, sometimes, or not yet on questions regarding a child's ability to perform a task. This study utilized a summed ASQ-3 score across all domains, with higher scores suggesting better development. The ASQ-3 constitutes as robust a detector of developmental delays among children as do the Parents' Evaluation of Developmental Status (PEDS) and Survey of Well-being of Young Children (SWYC) (26). A score lower than the mean ± SD was defined as the occurrence of “suspected developmental delay.” Items with an expected *a posteriori*/plausible value of reliability were above 0.99 for all domains. Test–retest reliability was high (0.95–0.99), as the agreement with the concurrent measure ranged from 0.29 to 0.89 (27).

### Responsive caregiving assessment

An infant-nurturing care questionnaire based on the Five Elements of Parenting Care Framework released by the WHO (13) was used to assess the level of early responsive caregiving for infants. The three items of the questionnaire were “on-demand feeding, smiling, and looking at the baby,” “offering infants skin-to-skin contact,” and “offering positive interaction.” Each item was scored from 0 to 3 according to its frequency (from “never” to “very often”). The scoring of this scale was based on the total score for all items (a higher score represented a better outcome). We defined “insufficient responsive caregiving” as the lowest tertile and the top two tertiles as “sufficient responsive caregiving.” Cronbach's *α* coefficient for the questionnaire was 0.864 (11).

### Covariates

A number of variables that are proposed to be related to children's neurodevelopment and maternal psychological stress have been determined to be potential confounders. We collected maternal sociodemographic information with questionnaires at 12–16 weeks of gestational age, including maternal educational level using a scale of 1 (i.e., middle school and below) to 4 (college and above), working status as employed (1) or unemployed (2), and annual family income in increments of ¥100,000 (1, <¥100,000; 4, ≥¥300,000). Information on maternal age at delivery (recorded in years), gestational age at birth (recorded in weeks), maternal parity, delivery mode, and infant sex were all retrieved from birth records. When the child was 2, 6, and 12 months of age, we also compiled information on feeding style (i.e., 1, exclusive breastfeeding; 2, mixed feeding; and 3, artificial feeding) and on primary caregivers (0, mother; 1, others).

### Statistical analysis

Analyses of descriptive statistics, multivariate logistic regression, and stratified logistic regression were conducted using IBM SPSS Statistics version 21.0, and the significance level was set at 0.05. Means ± standard deviations and proportions were employed to describe population characteristics. For missing values, we used multiple imputation models (the number of imputed datasets was 20), assuming that missing data were missing completely at random. Sociodemographic characteristics of the participants with or without assessment of the respective 12-month-old infants' early neurodevelopment were compared using Chi-squared and *t* tests. Correlations between scores of early developmental domains among children were additionally reported as Pearson correlation coefficients. We implemented multivariate logistic regression to analyze the associations between maternal psychological stress during pregnancy and children's neurodevelopment at 6 and 12 months of age.

To examine the potential modifying effects of responsive caregiving on the above associations, we executed Chi-squared tests to compare the rates of insufficient responsive caregiving between the high and relatively low maternal stress groups. We also compared the early development of infants at 6 (mean ± SD, 6.12 ± 0.22) and 12 (12.24 ± 0.55) months of age between the “insufficient responsive caregiving” and “sufficient responsive caregiving” groups. We then exploited models to test multiplicative interactions by the inclusion of an interaction term in the fully adjusted model (maternal psychological stress × responsive caregiving) and determined the effects of interactions. Analyses took place in three steps. First, model I consisted of examinations of maternal psychological stress and children's neurodevelopment with adjustments of sociodemographic data; model II included model I variables plus an interaction term (maternal psychological stress × responsive caregiving); and model III was model I stratified by the infant-nurturing care scores. The important confounding factors controlled in each model included maternal age at delivery, maternal educational level, household income, gestational weeks at birth, birth weight, and primary caregivers at 2 months of age. Considering the potential pathway(s) underlying infant feeding style in the causal relationship between maternal psychosocial stress during pregnancy and suspected developmental delay in infants, we assessed mediating effects with a sensitivity analysis rather than with adjusted confounders.

Conceptually, variables mediating infant feeding style are hypothesized to partially account for the relationship between a predictive variable of maternal stress in infants and an outcome variable of suspected developmental delay. To explore potential mediating effects, we performed a bootstrap analysis developed by Preacher and Hayes (28) using only those stress factors identified as significantly associated with suspected developmental delay of infants in main effects tested for mediation (i.e., total stress, subjective event stress, and general negative objective events stress during early pregnancy; and general negative objective events stress during late pregnancy). An indirect effect was considered significant if the 95% confidence interval (CI) calculated from the 5,000 bootstrap samples did not include zero. We evaluated the mediating effects of infant feeding style using the PROCESS plugin included in SPSS by adjusting for maternal age at delivery, educational level, household income, gestational weeks at birth, birth weight, and primary caregivers at 2 months of age.

## Results

### Descriptive statistics

Descriptive statistics are presented in Table 1. The final study population included 2,957 mother–child pairs, and the mean age of the mothers was 29.07 years (SD, 24.08). As for the socioeconomic status, 1,561 (43.3%) had completed at least college education, and 2,587 (71.8%) possessed a middle income and above (more than ¥100,000 per year). The prematurity rate was 7.3% and the mean birth weight was 3,327 g (SD, 504 g). As shown in Supplementary Table S1, participants who finished infant measurements using the ASQ possessed similar demographic characteristics compared to those lost to follow-up. After testing for normality, we observed that the total ASQ score was not normally distributed and that it ranged from 60 to 300 at 6 months of age and from 25 to 300 at 12 months of age—with average total scores of 220.3 (SD, 46.8) and 235.8 (SD, 44.9) at 6 and 12 months of age, respectively. The rate of suspected developmental delay ranged from 12.4% to 24.5%, with rates of developmental delay on communication, gross motor, fine motor, problem-solving, and personal–social skills of 13.3%, 18.7%, 16.8%, 17.1%, and 24.5% at 6 months of age (mean ± SD, 6.12 ± 0.22), respectively. At 12 months of age (mean ± SD, 12.24 ± 0.55), the rates of suspected developmental delay on the five domains were 19.7%, 14.4%, 15.3%, 18.0%, and 19.0%, respectively. Correlation analysis revealed that all scores for developmental domains were significantly correlated with one another; and that the Pearson correlation coefficients ranged from 0.29 to 0.57 and 0.26 to 0.61 at 6 and 12 months, respectively (see Supplementary Table S2 for details). The mean score for responsive caregiving at the age of 2 months was 7.39 ± 1.63, and the cutoff value for P30 was 5.

**Table 1:** Characteristics of participants and study variables (*N* = 3603).

Sociodemographic characteristics	*N* (%)
Maternal education	
Middle school and below	339 (9.4)
High school or same level	467 (13.0)
Junior college	1,214 (33.7)
College or beyond	1,561 (43.3)
Missing	22 (0.6)
Maternal employment status	
Employment	2,663 (73.9)
Unemployed	782 (21.7)
Missing	158 (4.4)
Household income per year	
<¥100,000	890 (24.7)
¥100,000–200,000	1,618 (44.9)
¥200,000–300,000	641 (17.8)
>¥300,000	328 (9.1)
Missing	126 (3.5)
Maternal parity	
Primiparous	1,859 (51.6)
Non-primiparous	1,478 (41.0)
Missing	266 (7.4)
Gestational age at birth, weeks^a^	39.09 (1.30)
Infant’s sex	
Male	1,848 (51.3)
Female	1,755 (48.7)
Delivery mode	
Vaginal	1,600 (44.4)
Cesarean	1,744 (48.4)
Missing	259 (7.2)
Feeding patterns for 2-month-old infants	
Exclusive breastfeeding	1,614 (44.8)
Mixed feeding	1,412 (39.2)
Artificial feeding	289 (8.0)
Missing	288 (8.0)
Feeding patterns for 6-month-old infants	
Breastfeeding or Mixed feeding	2,481 (83.9)
Artificial feeding	242 (8.2)
Missing	234 (7.9)
Feeding patterns for 12-month-old infants	
Breastfeeding or mixed feeding	309 (13.8)
Artificial feeding	1,758 (78.5)
Missing	172 (7.7)
Primary caregiver of 2-month-old infants	
Mother	2,810 (78.0)
Others	721 (20.0)
Missing	72 (2.0)
Maternal stress during pregnancy^b^	
At 12–16 weeks of pregnancy	
SE	89
OE1	111
OE2	53
OE3	74
Total	292
At 32–36 weeks of pregnancy	
SE	41
OE1	65
OE2	52
OE3	41
Total	197

SE, subjective events; OE1, positive objective events; OE2, general negative objective events; OE3, severe negative objective events.

^a^ Values are expressed as mean (standard deviation).

^b^ Values are expressed as 75th percentile.

### Maternal psychological stress during pregnancy and early neurodevelopment in infants

As shown in Table 2, high maternal subjective events (SE) stress during early pregnancy was associated with an increased risk of suspected developmental delay in problem-solving domains at 12 months of age [adjusted OR (aOR) = 1.51; 95% confidence interval (CI), 1.09–2.20] after adjusting for significant confounding factors. High objective events stress (OE2) during late pregnancy was also a risk factor for development in the personal–social domain at 12 months of age (aOR = 1.57; 95% CI, 1.13–2.19). However, total maternal stress during early pregnancy was associated with a reduced risk of suspected developmental delay in communication and gross motor domains at 6 months of age, and the adjusted ORs (95% CIs) were 0.68 (0.47–0.95) and 0.73 (0.54–0.92), respectively. We noted that high objective events stress (OE2) during early pregnancy was a protective factor with respect to development in communication and gross motor and fine motor domains at six months of age; and the adjusted ORs (95% CIs) were 0.55 (0.39–0.77), 0.67 (0.51–0.88), and 0.65 (0.49–0.87). Using sensitivity analysis, we tested the mediating effects of infant feeding style in the above analysis and observed no significant mediation effect of maternal psychological stress on neurodevelopment in infants with respect to infant feeding style (see Supplementary Table S3 for details).

**Table 2:** Multivariate logistic regression analysis of the association between maternal psychological stress and neurodevelopment in infants (model I).

Maternal stress		Communication	Gross motor	Fine motor	Problem-solving	Personal–social
		OR (95%CI)	OR (95%CI)	OR (95%CI)	OR (95%CI)	OR (95%CI)
Six months of age (*N* = 2,957)						
At 12–16 weeks of pregnancy	SE	0.77 (0.54–1.10)	0.76 (0.57–1.03)	0.86 (0.63–1.16)	0.84 (0.62–1.15)	0.76 (0.59–1.01)
	OE1	0.69 (0.48–1.03)	0.91 (0.68–1.21)	0.85 (0.635–1.15)	0.76 (0.56–1.04)	0.86 (0.66–1.11)
	OE2	0.55 (0.39–0.77)**	0.67 (0.51–0.88)**	0.65 (0.49–0.87)**	0.74 (0.55–1.01)	0.82 (0.64–1.03)
	OE3	0.85 (0.61–1.20)	0.91 (0.69–1.20)	0.99 (0.74–1.33)	0.86 (0.63–1.16)	0.95 (0.74–1.21)
	Total	0.68 (0.47–0.95)*	0.73 (0.54–0.92)*	0.81 (0.59–1.11)	0.76 (0.55–1.05)	0.82 (0.63–1.06)
At 32–36 weeks of pregnancy	SE	0.89 (0.65–1.22)	1.01 (0.77–1.33)	1.07 (0.80–1.42)	0.94 (0.71–1.25)	1.09 (0.85–1.39)
	OE1	0.99 (0.724–1.38)	0.97 (0.73–1.29)	1.05 (0.79–1.41)	1.02 (0.76–1.36)	0.91 (0.70–1.17)
	OE2	0.68 (0.46–0.99)*	0.71 (0.51–0.98)*	0.98 (0.71–1.35)	0.78 (0.56–1.09)	0.80 (0.61–1.07)
	OE3	1.02 (0.72–1.43)	1.17 (0.87–1.56)	1.19 (0.88–1.60)	1.07 (0.79–1.45)	1.12 (0.86–1.46)
	Total	0.93 (0.64–1.34)	0.93 (0.68–1.28)	1.10 (0.79–1.52)	0.84 (0.60–1.17)	0.83 (0.62–1.11)
12 months of age (*N* = 2239)						
At 12–16 weeks of pregnancy	SE	1.07 (0.79–1.45)	1.10 (0.79–1.54)	0.99 (0.71–1.40)	1.51 (1.09–2.20)*	0.91 (0.67–1.25)
	OE1	0.74 (0.53–1.01)	1.15 (0.83–1.61)	0.99 (0.69–1.40)	0.83 (0.60–1.16)	0.98 (0.73–1.33)
	OE2	0.73 (0.54–0.97)*	1.00 (0.74–1.36)	0.86 (0.63–1.17)	1.01 (0.75–1.34)	1.09 (0.83–1.43)
	OE3	1.16 (0.86–1.57)	0.99 (0.71–1.40)	0.99 (0.70–1.38)	1.13 (0.82–1.54)	1.02 (0.76–1.40)
	Total	0.89 (0.65–1.22)	0.94 (0.67–1.34)	0.93 (0.65–1.31)	1.12 (0.82–1.54)	1.04 (0.77–1.41)
At 32–36 weeks of pregnancy	SE	0.82 (0.60–1.12)	0.91 (0.64–1.30)	1.04 (0.85–1.22)	1.13 (0.82–1.55)	1.06 (0.77–1.45)
	OE1	0.85 (0.62–1.17)	1.27 (0.90–1.80)	0.99 (0.70–1.43)	1.23 (0.89–1.70)	1.35 (0.98–1.84)
	OE2	0.91 (0.65–1.29)	1.11 (0.76–1.61)	1.19 (0.81–1.74)	1.44 (0.99–2.02)	1.57 (1.13–2.19)**
	OE3	0.94 (0.67–1.31)	0.86 (0.59–1.25)	1.22 (0.85–1.76)	1.06 (0.75–1.49)	1.19 (0.86–1.64)
	Total	0.84 (0.59–1.20)	0.98 (0.66–1.45)	1.20 (0.95–2.10)	1.19 (0.83–1.70)	1.45 (0.99–2.02)

SE, subjective events; OE1, positive objective events; OE2, general negative objective events; OE3, severe negative objective events.

All of the models adjusted for maternal age at delivery, education level, household income, gestational weeks at birth, birth weight, and main caregivers at 2 months old.

Reference category: Low stress level, relatively normal neurodevelopment.

* P < 0.05; **P < 0.01.

### Modification effect of responsive caregiving at 2 months of age in the association between maternal stress during pregnancy and early development in infants

Higher maternal stress during pregnancy was not associated with insufficient responsive caregiving at two months of age. However, in the insufficient responsive caregiving group, the rates of suspected delay of neurodevelopment in infants at 6 (mean ± SD, 6.12 ± 0.22) and 12 (12.24 ± 0.55) months of age were significantly higher than in the sufficient group (*P* < 0.05) (Table 3).

**Table 3:** Associations between responsive caregiving and suspected developmental delay in early infant neurodevelopment.

		
		Communication		Gross motor		Fine motor		Problem-solving		Personal–social		Total score	
		*n* (%)	*χ* ^2^	*n* (%)	*χ* ^2^	*n* (%)	*χ* ^2^	*n* (%)	*χ* ^2^	*n* (%)	*χ* ^2^	*n* (%)	*χ* ^2^
Suspected developmental delay of early neurodevelopment in infants at 6 months of age													
Responsive caregiving at 2 months	Insufficient	134 (15.6)	5.35*	199 (23.1)	15.25***	167 (19.4)	5.83*	173 (20.1)	7.25**	244 (28.3)	9.43**	293 (34.0)	9.12**
	Sufficient	258 (12.4)		353 (16.9)		328 (15.7)		333 (16.0)		479 (23.0)		592 (28.4)	
Suspected developmental delay of infants’ early neurodevelopment in 12 months of age													
Responsive caregiving at 2 months	Insufficient	166 (24.7)	15.74***	116 (17.3)	5.90*	133 (19.8)	14.56***	143 (21.3)	7.19**	157 (23.4)	12.21***	336 (50.0)	13.40***
	Sufficient	271 (17.4)		207 (13.3)		209 (13.4)		257 (16.5)		265 (17.0)		647 (41.6)	

* P < 0.05; **P < 0.01; ***P < 0.001.

**Figure 2:**
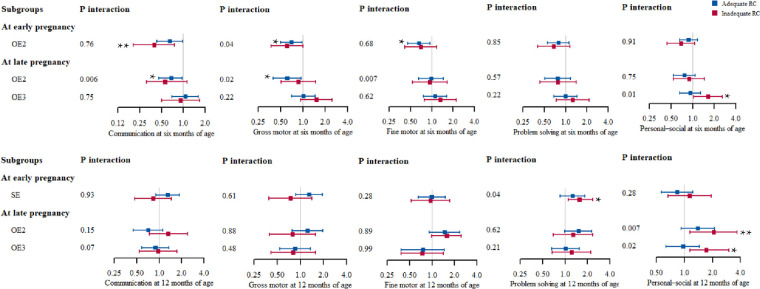
Forest plot of OR with 95% CIs of suspected developmental delay of infants grouped by maternal psychological stress and stratified by responsive caregiving (model III). The participants in low stress level group were used as a reference. All of the models adjusted for maternal age at delivery, education level, household income, gestational weeks at birth, birth weight, and main caregivers at 2 months of age. *P < 0.05; **P < 0.01. SE, subjective events; OE2, general negative objective events; OE3, severe negative objective events; RC, responsive caregiving.

As shown in Table 4, we uncovered an interaction effect between maternal psychological stress and responsive caregiving in the relationship between maternal psychological stress and infant neurodevelopment. We then conducted subgroup analysis to investigate the impacts of maternal psychological stress on child neurodevelopment as stratified by responsive caregiving, and discerned that early responsive caregiving moderated the relationships between maternal psychological stress and infant neurodevelopment. Maternal objective events (OE2) during early pregnancy (especially for children with sufficient early responsive caregiving) were associated with 61% (aOR = 0.39; 95% CI, 0.21–0.76) and 30% (aOR = 0.70; 95% CI, 0.50–0.98) diminutions in the risks regarding communication and gross motor domains at 6 months of age, respectively. Furthermore, OE2 during late pregnancy were associated with 32% (aOR = 0.68; 95% CI, 0.46–0.97) and 39% (aOR = 0.61; 95% CI, 0.40–0.94) reductions in the risks for communication and gross motor domains at 6 months of age, respectively. The significant risk association between maternal psychological stress and child neurodevelopment was restricted to the insufficient responsive caregiving group. For children with insufficient early responsive caregiving, we observed greater associations between the risk for maternal negative objective events (OE2) during late pregnancy and personal–social skills at 12 months of age (aOR = 2.06; 95% CI, 1.15–3.68). We similarly observed a greater association between the risk for maternal SE during early pregnancy and problem-solving at 12 months of age (aOR = 1.55; 95% CI, 1.11–2.34) for infants with insufficient early responsive caregiving (Figure 2).

**Table 4:** Multivariate logistic model of maternal psychological stress associations with infant development vis-à-vis the interactions between maternal stress and responsive caregiving at 2 months of age (model II).

	Communication	Gross motor	Fine motor	Problem-solving	Personal–social
	OR (95%CI)	OR (95%CI)	OR (95%CI)	OR (95%CI)	OR (95%CI)
Suspected developmental delay at 6 months of age					
OE2 during early pregnancy* Responsive caregiving	1.69 (0.79–3.61)	0.68 (0.50–0.95)*	1.01 (0.55–1.87)	1.23 (0.66–2.27)	1.33 (0.80–2.22)
OE2 during late pregnancy* Responsive caregiving	1.42 (1.24–1.74)**	1.69 (1.10–1.96)*	1.67 (1.24–2.12)**	1.04 (0.82–2.10)	0.89 (0.50–1.61)
OE3 during late pregnancy* Responsive caregiving	1.18 (0.58–2.42)	0.69 (0.38–1.25)	0.88 (0.47–1.65)	0.83 (0.44–1.56)	0.57 (0.33–0.95)*
Suspected developmental delay at 12 months of age					
SE during early pregnancy* Responsive caregiving	1.68 (0.87–2.25)	1.67 (0.80–2.49)	1.02 (0.49–2.10)	0.56 (0.34–0.91)*	0.70 (0.36–1.34)
OE2 during early pregnancy* Responsive caregiving	0.82 (0.44–1.52)	0.76 (0.39–1.48)	0.95 (0.48–1.85)	0.96 (0.52–1.80)	0.57 (0.36–0.80)**
OE2 during late pregnancy* Responsive caregiving	0.56 (0.27–1.14)	1.37 (0.61–2.06)	1.75 (0.78–2.91)	1.08 (0.52–2.24)	0.64 (0.32–0.73)**
OE3 during late pregnancy* Responsive caregiving	0.85 (0.43–1.71)	0.92 (0.41–2.14)	1.91 (0.87–2.17)	0.78 (0.38–1.61)	0.51 (0.25–0.95)*

SE, subjective events; OE2, general negative objective events; OE3, severe negative objective events.

All of the models adjusted for maternal age at delivery, education level, household income, gestational weeks at birth, birth weight, and main caregivers at 2 months of age.

* P <  0.05; **P < 0.01.

## Discussion

In the current study, we conducted a prospective cohort analysis to investigate the associations between maternal prenatal psychological stress during different gestational periods and neurodevelopment of children at 6 and 12 months of age and evaluated the modification effects of responsive caregiving in early infancy. We herein revealed that children whose mothers were exposed to higher levels of psychological stress during pregnancy were more likely to exhibit poor neurodevelopment in infancy, particularly those mothers who underwent high maternal subjective event stress in early pregnancy and high negative objective events stress in late pregnancy. We observed that sufficient responsive care in infancy moderated the relationships between subjective and objective event stress in mothers during pregnancy and the neurodevelopment of children at 12 months of age.

The data from the present study showed that maternal stress may harm neurodevelopment of children at 6 and 12 months of age and that the effect may depend upon the timing of the stress experienced. Specifically, high maternal SE stress during early pregnancy was associated with an increased risk of suspected developmental delay in the problem-solving domain, and this was consistent with previous studies (29). High negative objective events stress during late pregnancy was associated with an increased risk of suspected developmental delay in the personal–social domain, in accordance with previous studies showing that maternal prenatal stress was associated with socioemotional development in offspring (2, 30). Additionally, the development of the communication domain in infants was more vulnerable to maternal negative objective stress, congruent with a study depicting children exposed to antenatal depression as presenting with language delay at 2 years of age (5). According to a systematic review, prenatal stress was associated with a 66% increase in the risk of child socioemotional problems (2). Chan et al. also implicated parental stress exposures in the risk for offspring neurodevelopmental and neuropsychiatric disorders (31). Thus, psychosocial assessment and social support should be offered to pregnant women in early pregnancy, and they need to avoid experiencing stressful events during late pregnancy so as to prevent adverse neurodevelopmental outcomes in their infants.

A salient finding of the present study was the modification by responsive caregiving of the association between maternal psychological stress during pregnancy and infant neurodevelopment. Previous studies have indicated that combined exposures to maternal psychological stress and other environmental factors may exert more robust detrimental effects on childhood neurodevelopment. For example, Guo et al. ascertained that maternal combined exposure to stress and lead impaired infant neurodevelopment more profoundly than did a single exposure (32), and maternal prenatal stress appeared to exacerbate the deleterious effects of prenatal exposure to lead on toddlers' cognitive development (33). Wade et al. showed that positive postnatal experiences—particularly high levels of responsive parenting—protected children's social cognition against the deleterious effects of cumulative biomedical risk (34). Although extant studies have highlighted the necessity of evaluating the combined effects of multiple exposures on disease risk, we know of only a few investigations on the interactive effects of maternal psychological stress and responsive caregiving on early neurodevelopment in infants. In this study, early responsive caregiving moderated the relationships between maternal psychological stress and infant neurodevelopment, and significant associated risk was restricted to the insufficient responsive caregiving group. Our data thus underscore the importance of reducing maternal prenatal psychological stress and providing sufficient responsive caregiving in early infancy to prevent detrimental neurodevelopmental outcomes in children. These findings collectively suggest that we give greater attention to children born to mothers experiencing higher levels of stress during pregnancy, actively improve mothers' behaviors regarding rearing and nursing, reduce the adverse factors in the growth and development environment of infants, and attenuate the potential negative effects of psychological stress during pregnancy.

The mechanisms by which maternal psychological stress influence children's neurodevelopment are presumed to involve changes in infant brain development. Experimental evidence from animal models has revealed that prenatal exposure to maternal stress affected offspring neurodevelopment, neurocognitive function, cerebral processing, functional and structural brain connectivity involving the amygdala and prefrontal cortex, and the hypothalamic-pituitary-adrenal axis (35). Neuroimaging evidence also depicted alterations in brain structure and function in the frontal, temporal, and limbic areas in children born to mothers who experienced prenatal anxiety (36). Our most important finding was that responsive caregiving moderated the association between maternal psychological stress during pregnancy and neurodevelopment in infants. While the precise role of responsive caregiving requires further investigation, an interventional study showed that health of at-risk neonates was facilitated *via* training programs that build parents' cognitive and affective responsiveness (37). A longitudinal study also showed that the provision of early responsive caregiving was associated with enhanced physiological organization and resultant cognitive functioning over the first 10 years of life (38).

The present study was strengthened by its prospective cohort design, which facilitated the establishment of a temporal relationship between maternal psychological stress and childhood outcomes. Additionally, our approach specified vulnerable periods and indicated that mothers with high psychosocial stress during late pregnancy were more severely affected. Maternal psychological stress during pregnancy was divided into subjective events, positive objective events, general negative objective events, and severe negative objective events. This partitioning allowed the clear analysis of the association between different types of stress and the different domains of early neurodevelopment in infants. Our data thus provided evidence for the effect modification of responsive caregiving on infant neurodevelopment under maternal psychological stress during pregnancy.

However, despite attributing several important strengths to our study, we acknowledge some limitations. First, the participants in the present study were only enrolled from the urban area of Shanghai and the levels of education among mothers were relatively high (nearly 77% had completed college or beyond). This may have limited the extrapolation of the results, although we did control the educational levels of the mothers in the data analysis process. Second, our data reflected missing information, such as loss to follow-up. The missing data were assumed to be missing at random (i.e., the probability that a variable was missing depended only upon the available information), enabling the application of multiple imputations. Another limitation was that responsive caregiving in infancy was only assessed at 2 months of age, and therefore longer-lasting effects of responsive caregiving will need to be addressed in the future.

## Conclusions

Maternal psychological stress during pregnancy was predominantly associated with suspected developmental delay in infants at 6 and 12 months of age, and early responsive caregiving at 2 months moderated these relationships. Our findings indicate that improvements in early responsive caregiving levels to infants—especially when mothers were affected by negative life events during pregnancy—are necessary so as to avoid further disruption of infant development.

## Data Availability

The original contributions presented in the study are included in the article/**Supplementary Material**, further inquiries can be directed to the corresponding authors.
