# Supplementary material for: Longitudinal association between maternal psychological stress during pregnancy and infant neurodevelopment: The moderating effects of responsive caregiving
Source: Front Pediatr. 2022 Nov 18;10:1007507. doi: 10.3389/fped.2022.1007507 (PMC9715968; doi:10.3389/fped.2022.1007507)
Supplement: Supplementary file 1 [file Table1.docx]

Supplementary Material

# Supplementary Table

Table. Multivariate logistic model of the association between maternal stress and infant development and the interaction of maternal stress and responsive caregiving at 2-month-old

| Maternal stress |  |  |  |  | 6-month-old (N=2549) | |  |
| --- | --- | --- | --- | --- | --- | --- | --- |
|  |  | Responsive caregiving | Communication | Gross motor | Fine motor | Problem solving | Personal-social |
|  |  |  | OR (95%CI) | OR (95%CI) | OR (95%CI) | OR (95%CI) | OR (95%CI) |
| At 12–16 weeks of pregnancy | SE | S | 0.46 (0.20~1.05) | 0.60 (0.32~1.11) | 0.81 (0.43~1.50) | 0.55 (0.29~1.08) | 0.56 (0.33~1.01) |
|  |  | I | 0.73 (0.43~1.22) | 0.76 (0.50~1.16) | 0.88 (0.53~1.47) | 0.99 (0.65~1.50) | 0.83 (0.58~1.19) |
|  | SE * Responsive caregiving | | 1.13 (0.48~2.67) | 0.92 (0.48~1.78) | 0.77 (0.40~1.47) | 1.34 (0.66~2.71) | 1.12 (0.63~1.99) |
|  | OE1 | S | 0.41 (0.16~1.05) | 0.64 (0.32~1.27) | 0.70 (0.35~1.42) | 0.58 (0.28~1.20) | 1.05 (0.60~1.84) |
|  |  | I | 0.77 (0.46~1.27) | 0.93 (0.63~1.39) | 1.24 (0.77~2.00) | 0.93 (0.61~1.41) | 0.89 (0.63~1.27) |
|  | OE1 * Responsive caregiving | | 1.29 (0.50~3.32) | 1.04 (0.52~2.11) | 1.00 (0.49~2.04) | 1.31 (0.60~2.86) | 0.75 (0.42~1.33) |
|  | OE2 | S | 0.35 (0.16~0.79)* | 0.53 (0.30~0.95)* | 0.54 (0.29~1.00) | 0.57 (0.31~1.05) | 0.62 (0.37~1.04) |
|  |  | I | 0.64 (0.40~1.02) | 0.73 (0.50~1.07) | 0.73 (0.47~1.15) | 0.85 (0.58~1.26) | 0.90 (0.65~1.24) |
|  | OE2 * Responsive caregiving | | 1.16 (0.97~1.39) | 0.67 (0.46~0.98)* | 0.95 (0.62~1.46) | 1.14 (0.83~1.58) | 0.79 (0.50~1.25) |
|  | OE3 | S | 0.62 (0.29~1.32) | 0.84 (0.47~1.50) | 0.98 (0.54~1.78) | 0.75 (0.40~1.39) | 0.72 (0.42~1.24) |
|  |  | I | 0.98 (0.60~1.59) | 0.94 (0.63~1.41) | 1.18 (0.72~1.94) | 0.94 (0.62~1.43) | 0.93 (0.65~1.32) |
|  | OE3 * Responsive caregiving | | 1.14 (0.51~2.42) | 0.80 (00.44~1.46) | 0.74 (0.40~1.37) | 0.98 (0.51~1.88) | 0.95 (0.55~1.63) |
|  | Total | S | 0.41 (0.17~1.01) | 0.55 (0.28~1.08) | 0.74 (0.37~1.46) | 0.55 (0.26~1.13) | 0.62 (0.34~1.12) |
|  |  | I | 0.66 (0.39~1.11) | 0.61 (0.39~1.01) | 0.80 (0.51~1.24) | 0.78 (0.50~1.21) | 0.80 (0.56~1.15) |
|  | Total stress * Responsive caregiving | | 1.21 (0.47~3.16) | 0.84 (0.41~1.74) | 0.81 (0.40~1.64) | 1.26 (0.57~2.78) | 1.07 (0.58~1.98) |
| At 32–36 weeks of pregnancy | SE | S | 0.45 (0.20~1.01) | 0.58 (0.32~1.08) | 0.78 (0.42~1.45) | 1.03 (0.58~1.80) | 1.45 (0.87~2.41) |
|  |  | I | 0.71 (0.46~1.14) | 0.76 (0.51~1.13) | 0.90 (0.59~1.36) | 0.82 (0.54~1.23) | 0.91 (0.64~1.29) |
|  | SE * Responsive caregiving | | 0.73 (0.39~1.34) | 0.63 (0.38~1.06) | 0.69 (0.40~1.17) | 0.77 (0.48~1.33) | 0.73 (0.46~1.16) |
|  | OE1 | S | 0.45 (0.18~1.12) | 0.64 (0.33~1.26) | 0.72 (0.36~1.43) | 0.79 (0.43~1.47) | 0.76 (0.44~1.31) |
|  |  | I | 0.92 (0.59~1.45) | 0.91 (0.62~1.36) | 1.19 (0.79~1.78) | 1.11 (0.75~1.66) | 0.95 (0.67~1.34) |
|  | OE1 * Responsive caregiving | | 1.03 (0.52~2.04) | 1.01 (0.56~1.83) | 1.07 (0.59~1.94) | 1.14 (0.62~2.08) | 1.13 (0.66~1.93) |
|  | OE2 | S | 0.33 (0.15~0.73)** | 0.52 (0.29~0.92)* | 0.52 (0.28~0.95)* | 0.67 (0.34~1.30) | 1.00 (0.57~1.78) |
|  |  | I | 0.64 (0.37~1.10) | 0.59 (0.38~1.02) | 0.88 (0.55~1.40) | 0.76 (0.48~1.21) | 0.71 (0.47~1.06) |
|  | OE2 * Responsive caregiving | | 1.65 (1.22~2.24)** | 1.70 (1.22~2.37)* | 1.54 (1.12~2.12)** | 1.07 (1.00~1.13) | 0.72 (0.30~1.72) |
|  | OE3 | S | 0.60 (0.28~1.28) | 0.81 (0.45~1.45) | 0.94 (0.52~1.72) | 1.05 (0.57~1.94) | 0.99 (0.69~1.42) |
|  |  | I | 1.05 (0.66~1.68) | 1.05 (0.70~1.57) | 1.27 (0.84~1.93) | 1.22 (0.81~1.84) | 1.73 (1.01~2.97)* |
|  | OE3 * Responsive caregiving | | 0.65 (0.42~1.01) | 1.02 (0.64~1.64) | 0.83 (0.52~1.31) | 0.75 (0.51~1.10) | 0.55 (0.36~0.85)** |
|  | Total | S | 0.43 (0.17~1.06) | 0.55 (0.28~1.08) | 0.75 (0.38~1.47) | 0.76 (0.39~1.49) | 1.03 (0.57~1.83) |
|  |  | I | 0.91 (0.54~1.53) | 0.89 (0.57~1.40) | 1.26. (0.81~1.97) | 0.98 (0.62~1.55) | 0.78 (0.52~1.17) |
|  | Total stress * Responsive caregiving | | 1.26 (0.54~2.94) | 0.85 (0.43~1.65) | 1.05 (0.54~2.06) | 1.15 (0.56~2.36) | 0.81 (0.44~1.49) |
|  |  |  |  |  | 12-month-old (N=2239) | |  |
| At 12–16 weeks of pregnancy | SE | S | 0.77 (0.42~1.41) | 0.73 (0.36~1.48) | 0.94 (0.47~1.88) | 1.46 (0.97~2.26) | 1.23 (0.69~2.19) |
|  |  | I | 0.73 (0.43~1.22) | 1.35 (0.87~2.10) | 1.27. (0.80~2.03) | 1.46 (1.10~2.64)* | 1.12 (0.60~2.08) |
|  | SE * Responsive caregiving | | 0.94 (0.63~1.41) | 0.51 (0.26~1.01) | 1.43 (0.71~2.87) | 0.51 (0.27~0.96)* | 0.83 (0.57~1.20) |
|  | OE1 | S | 0.73 (0.36~1.48) | 1.82 (0.88~3.75) | 1.31 (0.61~2.78) | 1.03 (0.51~2.06) | 0.71 (0.35~1.45) |
|  |  | I | 0.77 (0.46~1.27) | 0.87 (0.55~1.37) | 1.00 (0.62~1.60) | 0.75 (0.48~1.19) | 0.75 (0.48~1.19) |
|  | OE1 * Responsive caregiving | | 0.98 (0.52~1.86) | 1.28 (0.64~2.56) | 0.89 (0.44~1.81) | 0.77 (0.42~1.42) | 0.54 (0.29~1.01) |
|  | OE2 | S | 0.82 (0.46~1.45) | 1.17 (0.62~2.24) | 1.16. (0.60~2.22) | 1.00 (0.55~1.81) | 1.63 (0.93~2.88) |
|  |  | I | 0.63 (0.40~1.02) | 0.77 (0.50~1.18) | 0.92 (0.59~1.43) | 1.11 (0.75~1.65) | 1.10 (0.75~1.65) |
|  | OE2 * Responsive caregiving | | 0.59 (0.34~1.03) | 0.65 (0.36~1.17) | 0.65 (0.36~1.19) | 0.76 (0.44~1.31) | 0.48 (0.28~0.80)** |
|  | OE3 | S | 1.09 (0.60~1.98) | 1.35 (0.69~2.63) | 0.67 (0.32~1.41) | 0.77 (0.40~1.47) | 1.10 (0.60~1.99) |
|  |  | I | 1.03 (0.68~1.58) | 0.85 (0.53~1.37) | 0.86 (0.52~1.41) | 1.21 (0.78~1.85) | 1.21 (0.78~1.85) |
|  | OE3 * Responsive caregiving | | 0.63 (0.34~1.17) | 0.62 (0.31~1.24) | 0.82 (0.37~1.80) | 1.03 (0.51~2.05) | 0.58 (0.31~1.10) |
|  | Total | S | 0.66 (0.34~1.29) | 1.23 (0.64~2.65) | 0.69 (0.31~1.52) | 0.92 (0.47~1.78) | 0.96 (0.51~1.81) |
|  |  | I | 0.83 (0.54~1.29) | 0.90 (0.56~1.44) | 1.04 (0.64~1.68) | 1.25 (0.81~1.92) | 1.25 (0.81~1.92) |
|  | Total stress * Responsive caregiving | | 0.86 (0.42~1.76) | 0.69 (0.34~1.41) | 1.10 (0.49~2.47) | 0.97 (0.49~1.94) | 0.80 (0.41~1.54) |
| At 32–36 weeks of pregnancy | SE | S | 1.07 (0.59~1.95) | 1.07 (0.53~2.15) | 1.36 (0.66~2.81) | 1.35 (0.70~2.60) | 0.91 (0.48~1.72) |
|  |  | I | 0.66 (0.43~1.01) | 0.91 (0.57~1.47) | 1.23 (0.74~2.04) | 0.96 (0.61~1.51) | 0.96 (0.61~1.51) |
|  | SE * Responsive caregiving | | 1.28 (0.79~2.06) | 0.88 (0.46~1.65) | 0.79 (0.42~1.50) | 0.82 (0.46~1.48) | 0.85 (0.48~1.52) |
|  | OE1 | S | 1.00 (0.53~1.87) | 1.60 (0.77~3.31) | 1.47 (0.70~3.09) | 1.20 (0.61~2.37) | 0.86 (0.44~1.69) |
|  |  | I | 0.92 (0.59~1.45) | 1.54 (0.98~2.43) | 0.86 (0.51~1.44) | 1.23 (0.80~1.91) | 1.23 (0.80~1.91) |
|  | OE1 * Responsive caregiving | | 0.80 (0.49~1.30) | 1.06 (0.55~2.07) | 0.58 (0.28~1.20) | 1.04 (0.53~2.02) | 1.12 (0.59~2.13) |
|  | OE2 | S | 1.29 (0.67~2.47) | 0.78 (0.36~1.70) | 0.69 (0.30~1.62) | 1.44 (0.71~2.90) | 1.48 (0.93~2.36) |
|  |  | I | 0.64 (0.37~1.10) | 1.26 (0.77~2.06) | 1.29 (0.75~2.21) | 1.48 (0.93~2.36) | 2.62 (1.34~4.93)* |
|  | OE2 * Responsive caregiving | | 1.23 (0.61~2.49) | 1.07 (0.63~1.83) | 0.97 (0.67~1.41) | 0.98 (0.94~1.02) | 0.30 (0.13~0.66)** |
|  | OE3 | S | 1.06 (0.56~2.01) | 0.97 (0.46~2.05) | 0.87 (0.39~1.92) | 1.45. (0.73~2.89) | 0.86 (0.53~1.38) |
|  |  | I | 0.83 (0.52~1.33) | 0.73 (0.44~1.22) | 1.04 (0.61~1.76) | 0.86 (0.53~1.38) | 2.11 (1.10~4.05)* |
|  | OE3 * Responsive caregiving | | 0.99 (0.65~1.51) | 0.52 (0.26~1.06) | 0.60 (0.35~1.02) | 0.76 (0.51~1.14) | 0.43 (0.19~0.93)* |
|  | Total | S | 0.68 (0.34~1.35) | 1.11 (0.51~2.39) | 0.80 (0.35~1.85) | 1.08 (0.53~2.22) | 1.04 (0.53~2.07) |
|  |  | I | 0.90 (0.54~1.48) | 1.04 (0.62~1.75) | 1.44 (0.83~2.48) | 0.88 (0.53~1.46) | 0.88 (0.53~1.46) |
|  | Total stress * Responsive caregiving | | 0.77 (0.37~1.60) | 0.98 (0.45~2.14) | 1.32 (0.56~3.08) | 0.86 (0.40~1.85) | 1.08 (0.55~2.13) |

Note: SE (subjective events), OE2 (general negative objective events), OE3 (severe negative objective events); S (sufficient responsive caregiving), I (insufficient responsive caregiving); * P < 0.05; ** P < 0.01.

All of the models adjusted for maternal age at delivery, education level, household income, gestational weeks at birth, birth weight, main caregivers at 2 months old, and feeding style.
