# Supplementary material for: Longitudinal association between maternal psychological stress during pregnancy and infant neurodevelopment: The moderating effects of responsive caregiving
Source: Front Pediatr. 2022 Nov 18;10:1007507. doi: 10.3389/fped.2022.1007507 (PMC9715968; doi:10.3389/fped.2022.1007507)
Supplement: Supplementary file 2 [file Table2.docx]

Supplementary Material

# Supplementary Table 1

Table S1. Socio-demographic characteristics of the participants by whom with or without complete follow-up data.

|  | Participants with complete information (N=2239), N (%) | Participants lacked complete information (N=1497), N (%) | *P* value |
| --- | --- | --- | --- |
| Maternal education |  |  |  |
| Middle school and below | 157 (7.0) | 115 (7.7) | 0.71 |
| High school or same level | 275 (12.3) | 169 (11.3) |  |
| Junior college | 782 (34.9) | 545 (36.4) |  |
| College and above | 1025 (45.8) | 668 (44.6) |  |
| Maternal working status |  |  |  |
| Employment | 1782 (79.6) | 1154 (77.1) | 0.07 |
| Unemployed | 457 (20.4) | 343 (22.9) |  |
| Household income per year (RMB) |  |  |  |
| <100,000 | 511 (22.8) | 365 (24.4) | 0.32 |
| 100,000–200,000 | 1115 (49.8) | 691 (46.1) |  |
| 200,000–300,000 | 414 (18.5) | 308 (20.6) |  |
| >300,000 | 119 (8.9) | 133 (8.9) |  |
| Maternal parity |  |  |  |
| Primiparous | 1258 (56.2) | 820 (54.8) | 0.39 |
| Non-primiparous | 981 (43.8) | 677 (45.2) |  |
| Gestational age at birth, weeks^a^ | 39.06±1.25 | 39.12±1.24 | 0.26 |
| Infants sex |  |  |  |
| Male | 1108 (49.5) | 772 (51.6) | 0.30 |
| Female | 1131 (50.5) | 725 (48.4) |  |
| Delivery mode |  |  |  |
| Vaginal | 1043 (46.6) | 720 (48.1) | 0.36 |
| Cesarean | 1196 (53.4) | 777 (51.9) |  |

a. Values are expressed as mean ± SD

# Supplementary Table 2

Table S2. Pearson correlation coefficients of early developmental domains among children.

|  |  |  | At six months of age | |  |  |  |  | At 12 months of age | |  |
| --- | --- | --- | --- | --- | --- | --- | --- | --- | --- | --- | --- |
|  | Communication | Gross motor | Fine motor | Problem solving | Personal-social |  | Communication | Gross motor | Fine motor | Problem solving | Personal-social |
| Communication at six mo | 1.00 |  |  |  |  |  |  |  |  |  |  |
| Gross motor at six mo | 0.29** | 1.00 |  |  |  |  |  |  |  |  |  |
| Fine motor at six mo | 0.34** | 0.45** | 1.00 |  |  |  |  |  |  |  |  |
| Problem solving at six mo | 0.32** | 0.46** | 0.57** | 1.00 |  |  |  |  |  |  |  |
| Personal-social at six mo | 0.31** | 0.57** | 0.45** | 0.57** | 1.00 |  |  |  |  |  |  |
| Communication at 12 mo | 0.22** | 0.08** | 0.24** | 0.20** | 0.15** |  | 1.00 |  |  |  |  |
| Gross motor at 12 mo | 0.09** | 0.30** | 0.19** | 0.21** | 0.21** |  | 0.26** | 1.00 |  |  |  |
| Fine motor at 12 mo | 0.18** | 0.17** | 0.23** | 0.26** | 0.19** |  | 0.44** | 0.39** | 1.00 |  |  |
| Problem solving at 12 mo | 0.19** | 0.16** | 0.28** | 0.27** | 0.24** |  | 0.46** | 0.36** | 0.59** | 1.00 |  |
| Personal-social at 12 mo | 0.18** | 0.22** | 0.27** | 0.29** | 0.27** |  | 0.45** | 0.40** | 0.52** | 0.61** | 1.00 |

*Note:* **P* < 0.05, ⁎⁎*P* < 0.01.

# Supplementary Table 3

Table S3. Mediating effect of infant feeding style on maternal psychological stress and neurodevelopment in infants

| **Maternal stress** | | |  | **Suspected developmental delay at six months of age** | | | |
| --- | --- | --- | --- | --- | --- | --- | --- |
|  |  |  |  | **Communication** | **Gross motor** | **Fine motor** | **Personal-social** |
| **At 12-16 weeks of pregnancy** | **OE2** | **Total effect** | **β (95% CI)** | -0.001 (-0.009,0.007) | 0.002 (-0.011,0.011) | -0.002 (-0.012,0.009) |  |
|  |  | **Direct effect** | **β (95% CI)** | -0.001 (-0.009,0.007) | 0.003 (-0.011,0.011) | -0.002 (-0.012,0.009) |  |
|  |  | **Indirect effect** | **BootSE** | 0.0002 | 0.0005 | 0.0003 |  |
|  | **Total** | **Total effect** | **β (95% CI)** | -0.000 (-0.003,0.002) | 0.002 (-0.002,0.005) |  |  |
|  |  | **Direct effect** | **β (95% CI)** | -0.000 (-0.003,0.002) | 0.002 (-0.002,0.005) |  |  |
|  |  | **Indirect effect** | **BootSE** | 0.0001 | 0.0002 |  |  |
|  |  |  |  | **Suspected developmental delay at 12 months of age** | | | |
| **At 12-16 weeks of pregnancy** | **OE2** | **Total effect** | **β (95% CI)** | -0.006 (-0.016,0.003) |  |  |  |
|  |  | **Direct effect** | **β (95% CI)** | -0.007 (-0.016,0.003) |  |  |  |
|  |  | **Indirect effect** | **BootSE** | 0.0005 |  |  |  |
| **At 32-36 weeks of pregnancy** | **OE2** | **Total effect** | **β (95% CI)** |  |  |  | -0.024 (-0.037, -0.011) |
|  |  | **Direct effect** | **β (95% CI)** |  |  |  | -0.024 (-0.038,0.011) |
|  |  | **Indirect effect** | **BootSE** |  |  |  | 0.0004 |

*Note:* All models were adjusted for maternal age at delivery, education level, household income, gestational weeks at birth, birth weight, and main caregivers at two months of age.

# Supplementary Table 4

Table S4. Multivariate logistic model of maternal psychological stress associations with infant development vis-à-vis the interactions between maternal stress and responsive caregiving at two months of age. (Model II and Model III)

| **Maternal stress** |  |  | **Suspected developmental delay at six months of age** | | | | |
| --- | --- | --- | --- | --- | --- | --- | --- |
|  |  | Responsive caregiving | Communication | Gross motor | Fine motor | Problem solving | Personal-social |
|  |  |  | OR (95%CI) | OR (95%CI) | OR (95%CI) | OR (95%CI) | OR (95%CI) |
| At 12–16 weeks of pregnancy | SE | S | 0.92 (0.60–1.41) | 0.80 (0.55–1.15) | 0.86 (0.58–1.26) | 0.98 (0.68–1.42) | 0.86 (0.62–1.18) |
|  |  | I | 0.52 (0.26–1.05) | 0.71 (0.42–1.19) | 0.86 (0.50–1.48) | 0.57 (0.32–1.04) | 0.61 (0.37–1.00) |
|  | SE * Responsive caregiving | | 1.19 (0.57–2.46) | 0.79 (0.45–1.39) | 0.68 (0.38–1.21) | 1.10 (0.59–2.05) | 0.94 (0.57–1.58) |
|  | OE1 | S | 0.70 (0.45–1.09) | 0.84 (0.59–1.19) | 0.90 (0.62–1.30) | 0.85 (0.59–1.23) | 0.82 (0.60–1.11) |
|  |  | I | 0.60 (0.30–1.21) | 1.14 (0.68–1.89) | 0.82 (0.46–1.45) | 0.58 (0.31–1.08) | 1.08 (0.67–1.74) |
|  | OE1 * Responsive caregiving | | 0.81 (0.40–1.66) | 0.58 (0.34–1.00) | 0.77 (0.43–1.38) | 0.99 (0.53–1.87) | 0.62 (0.38–1.01) |
|  | OE2 | S | 0.65 (0.43–0.97)* | 0.70 (0.50–0.98)* | 0.66 (0.46–0.94) | 0.79 (0.56–1.12) | 0.87 (0.66–1.16) |
|  |  | I | 0.39 (0.21–0.76) | 0.61 (0.37–1.00) | 0.70 (0.42–1.17) | 0.68 (0.40–1.15) | 0.69 (0.44–1.07) |
|  | OE2 * Responsive caregiving | | 1.69 (0.79–3.61) | 0.68 (0.50–0.95)* | 1.01 (0.55–1.87) | 1.23 (0.66–2.27) | 1.33 (0.80–2.22) |
|  | OE3 | S | 0.89 (0.59–1.36) | 0.86 (0.60–1.22) | 0.92 (0.63–1.33) | 0.87 (0.60–1.26) | 0.93 (0.68–1.26) |
|  |  | I | 0.70 (0.38–1.29) | 0.90 (0.56–1.46) | 1.02 (0.62–1.69) | 0.76 (0.45–1.30) | 0.87 (0.55–1.36) |
|  | OE3 * Responsive caregiving | | 0.87 (0.46–1.63) | 0.65 (0.39–1.09) | 0.59 (0.35–1.00) | 0.78 (0.45–1.36) | 0.72 (0.45–1.13) |
|  | Total | S | 0.69 (0.44–1.09) | 0.60 (0.41–1.00) | 0.74 (0.50–1.09) | 0.76 (0.52–1.12) | 0.80 (0.58–1.11) |
|  |  | I | 0.64 (0.32–1.26) | 1.09 (0.65–1.80) | 1.00 (0.58–1.73) | 0.74 (0.41–1.33) | 0.91 (0.56–1.47) |
|  | Total stress * Responsive caregiving | | 0.81 (0.39–1.67) | 0.46 (0.26–0.80) | 0.56 (0.31–1.00) | 0.74 (0.40–1.36) | 0.69 (0.42–1.13) |
| At 32–36 weeks of pregnancy | SE | S | 0.80 (0.53–1.20) | 0.82 (0.58–1.16) | 0.90 (0.63–1.30) | 0.85 (0.60–1.21) | 0.92 (0.68–1.25) |
|  |  | I | 1.06 (0.61–1.84) | 1.40 (0.88–2.23) | 1.42 (0.86–2.33) | 1.07 (0.65–1.77) | 1.51 (0.98–2.34) |
|  | SE * Responsive caregiving | | 0.67 (0.40–1.13) | 0.54 (0.35–1.01) | 0.59 (0.37–0.92) | 0.71 (0.45–1.13) | 0.64 (0.43–0.95) |
|  | OE1 | S | 1.07 (0.72–1.59) | 0.97 (0.68–1.38) | 1.13 (0.79–1.62) | 1.15 (0.81–1.63) | 0.98 (0.72–1.33) |
|  |  | I | 0.93 (0.52–1.68) | 0.94 (0.57–1.54) | 0.87 (0.51–1.49) | 0.71 (0.41–1.23) | 0.81 (0.51–1.30) |
|  | OE1 * Responsive caregiving | | 0.92 (0.52–1.63) | 0.72 (0.44–1.16) | 0.87 (0.53–1.44) | 1.07 (0.64–1.80) | 0.92 (0.59–1.45) |
|  | OE2 | S | 0.68 (0.46–0.97)* | 0.61 (0.40–0.94)* | 0.98 (0.66–1.46) | 0.77 (0.51–1.17) | 0.77 (0.54–1.09) |
|  |  | I | 0.58 (0.31–1.12) | 0.87 (0.51–1.49) | 0.93 (0.53–1.64) | 0.78 (0.44–1.40) | 0.89 (0.54–1.47) |
|  | OE2 * Responsive caregiving | | 1.42 (1.24–1.74)** | 1.69 (1.10–1.96)* | 1.67 (1.24–2.12)** | 1.04 (0.82–2.10) | 0.89 (0.50–1.61) |
|  | OE3 | S | 1.06 (0.70–1.61) | 1.01 (0.70–1.46) | 1.12 (0.77–1.63) | 1.00 (0.68–1.45) | 0.92 (0.66–1.28) |
|  |  | I | 0.91 (0.50–1.67) | 1.51 (0.93–2.48) | 1.32 (0.78–2.22) | 1.26 (0.74–2.14) | 1.64 (1.03–2.60)* |
|  | OE3 * Responsive caregiving | | 1.18 (0.58–2.42) | 0.69 (0.38–1.25) | 0.88 (0.47–1.65) | 0.83 (0.44–1.56) | 0.57 (0.33–0.95)* |
|  | Total | S | 1.02 (0.65–1.60) | 0.88 (0.58–1.32) | 1.11 (0.74–1.66) | 0.85 (0.56–1.29) | 0.81 (0.56–1.15) |
|  |  | I | 0.80 (0.41–1.55) | 0.95 (0.55–1.63) | 0.97 (0.55–1.71) | 0.72 (0.39–1.32) | 1.67 (0.99–2.65) |
|  | Total stress * Responsive caregiving | | 1.06 (0.53–2.13) | 0.67 (0.37–1.19) | 0.83 (0.46–1.49) | 0.91 (0.48–1.71) | 0.77 (0.45–1.32) |
|  |  |  | **Suspected developmental delay at 12 months of age** | | | | |
| At 12–16 weeks of pregnancy | SE | S | 1.29 (0.90–1.86) | 1.29 (0.87–1.93) | 0.99 (0.65–1.51) | 1.25 (0.86–1.83) | 0.84 (0.57–1.24) |
|  |  | I | 0.83 (0.47–1.46) | 0.74 (0.39–1.41) | 0.95 (0.52–1.72) | 1.55 (1.11–2.34)* | 1.14 (0.67–1.93) |
|  | SE * Responsive caregiving | | 1.68 (0.87–2.25) | 1.67 (0.80–2.49) | 1.02 (0.49–2.10) | 0.56 (0.34–0.91)* | 0.70 (0.36–1.34) |
|  | OE1 | S | 0.78 (0.53–1.14) | 0.96 (0.64–1.44) | 0.94 (0.63–1.42) | 0.82 (0.55–1.21) | 1.05 (0.73–1.52) |
|  |  | I | 0.77 (0.40–1.45) | 1.92 (1.00–3.58) | 1.29 (0.70–2.39) | 1.01 (0.55–1.84) | 0.94 (0.52–1.69) |
|  | OE1 * Responsive caregiving | | 0.74 (0.39–1.41) | 0.49 (0.26–1.00) | 0.57 (0.30–1.07) | 0.59 (0.32–1.11) | 0.77 (0.42–1.40) |
|  | OE2 | S | 0.70 (0.49–1.00) | 0.93 (0.64–1.35) | 0.86 (0.58–1.26) | 1.01 (0.71–1.44) | 0.97 (0.69–1.36) |
|  |  | I | 0.93 (0.55–1.57) | 1.21 (0.68–2.15) | 0.90 (0.51–1.58) | 1.01 (0.59–1.72) | 1.35 (0.82–2.23) |
|  | OE2 * Responsive caregiving | | 0.82 (0.44–1.52) | 0.76 (0.39–1.48) | 0.95 (1.48–1.85) | 0.96 (0.52–1.80) | 0.57 (0.36–0.80)** |
|  | OE3 | S | 1.20 (0.83–1.72) | 0.82 (0.54–1.24) | 1.07 (0.71–1.61) | 1.19 (0.82–1.74) | 0.99 (0.68–1.43) |
|  |  | I | 1.01 (0.58–1.76) | 1.41 (0.78–2.57) | 0.81 (0.44–1.48) | 0.93 (0.53–1.64) | 1.12 (0.66–1.91) |
|  | OE3 * Responsive caregiving | | 0.72 (0.42–1.25) | 0.54 (0.29–1.00) | 0.86 (0.46–1.60) | 0.80 (0.45–1.42) | 0.62 (0.36–1.08) |
|  | Total | S | 1.01 (0.70–1.48) | 0.87 (0.57–1.33) | 0.98 (0.64–1.48) | 1.18 (0.81–1.73) | 1.10 (0.76–1.59) |
|  |  | I | 0.78 (0.43–1.43) | 1.17 (0.62–2.21) | 0.83 (0.44–1.56) | 1.02 (0.57–1.84) | 1.01 (0.58–1.78) |
|  | Total stress * Responsive caregiving | | 0.87 (0.47–1.60) | 0.65 (0.34–1.25) | 0.80 (0.41–1.55) | 0.76 (0.42–1.38) | 0.71 (0.40–1.26) |
| At 32–36 weeks of pregnancy | SE | S | 0.70 (0.46–1.05) | 0.83 (0.53–1.29) | 1.86 (1.00–2.87) | 1.03 (0.69–1.53) | 1.12 (0.76–1.65) |
|  |  | I | 1.06 (0.61–1.82) | 1.03 (0.56–1.92) | 0.93 (0.52–1.68) | 1.30 (0.74–2.29) | 0.87 (0.50–1.51) |
|  | SE * Responsive caregiving | | 0.52 (0.31–0.87) | 0.72 (0.40–1.27) | 0.92 (0.54–1.57) | 0.70 (0.42–1.16) | 0.90 (0.54–1.50) |
|  | OE1 | S | 0.77 (0.51–1.15) | 1.29 (0.85–1.97) | 0.86 (0.55–1.37) | 1.17 (0.79–1.74) | 1.60 (1.09–2.35) |
|  |  | I | 1.08 (0.61–1.92) | 1.32 (0.70–2.50) | 1.27 (0.69–2.31) | 1.38 (0.77–2.48) | 0.97 (0.54–1.73) |
|  | OE1 * Responsive caregiving | | 0.56 (0.32–1.00) | 0.86 (0.47–1.56) | 0.49 (0.27–1.02) | 0.73 (0.42–1.26) | 1.05 (0.61–1.82) |
|  | OE2 | S | 0.71 (0.45–1.12) | 1.24 (0.79–1.94) | 1.46 (0.91–2.35) | 1.51 (0.99–2.29) | 1.40 (0.93–2.10) |
|  |  | I | 1.32 (0.73–2.39) | 0.79 (0.40–1.57) | 0.75 (0.39–1.47) | 1.28 (0.70–2.37) | 2.06 (1.15–3.69)* |
|  | OE2 * Responsive caregiving | | 0.56 (0.27–1.14) | 1.37 (0.61–2.06) | 1.75 (0.78–2.91) | 1.08 (0.52–2.24) | 0.64 (0.32–0.73)** |
|  | OE3 | S | 0.89 (0.58–1.35) | 0.85 (0.54–1.35) | 1.55 (0.99–2.45) | 1.01 (0.66–1.54) | 0.97 (0.64–1.46) |
|  |  | I | 0.97 (0.54–1.73) | 0.81 (0.41–1.58) | 0.74 (0.39–1.40) | 1.23 (0.68–2.22) | 1.72 (1.15–3.02)* |
|  | OE3 * Responsive caregiving | | 0.85 (0.43–1.71) | 0.92 (0.41–2.14) | 1.91 (0.87–2.17) | 0.78 (0.38–1.61) | 0.51 (0.25–0.95)* |
|  | Total | S | 0.85 (0.54–1.34) | 0.94 (0.58–1.54) | 2.15 (0.99–3.40) | 1.14 (0.73–1.77) | 1.67 (1.00–2.51) |
|  |  | I | 0.78 (0.41–1.46) | 1.05 (0.53–2.09) | 0.71 (0.36–1.42) | 1.20 (0.64–2.24) | 0.95 (0.51–1.75) |
|  | Total stress * Responsive caregiving | | 0.72 (0.37–1.39) | 0.77 (0.38–1.57) | 1.34 (0.66–2.72) | 0.76 (0.40–1.44) | 1.11 (0.60–2.07) |

*Note:* SE (subjective events), OE2 (general negative objective events), OE3 (severe negative objective events); S (sufficient responsive caregiving), I (insufficient responsive caregiving); * P < 0.05; ** P < 0.01. All models were adjusted for maternal age at delivery, education level, household income, gestational weeks at birth, birth weight, and main caregivers at two months of age.
